# Data cleaning process for HIV-indicator data extracted from DHIS2 national reporting system: a case study of Kenya

**DOI:** 10.1186/s12911-020-01315-7

**Published:** 2020-11-13

**Authors:** Milka Bochere Gesicho, Martin Chieng Were, Ankica Babic

**Affiliations:** 1 Department of Information Science and Media Studies, University of Bergen, Bergen, Norway; 2 Vanderbilt University Medical Center, Nashville, USA; 3 Department of Biomedical Engineering, Linköping University, Linköping, Sweden; 4 Institute of Biomedical Informatics, Moi University, Eldoret, Kenya

**Keywords:** Data-cleaning, dhis2, HIV-indicators, Data management

## Abstract

**Background:**

The District Health Information Software-2 (DHIS2) is widely used by countries for national-level aggregate reporting of health-data. To best leverage DHIS2 data for decision-making, countries need to ensure that data within their systems are of the highest quality. Comprehensive, systematic, and transparent data cleaning approaches form a core component of preparing DHIS2 data for analyses. Unfortunately, there is paucity of exhaustive and systematic descriptions of data cleaning processes employed on DHIS2-based data. The aim of this study was to report on methods and results of a systematic and replicable data cleaning approach applied on HIV-data gathered within DHIS2 from 2011 to 2018 in Kenya, for secondary analyses.

**Methods:**

Six programmatic area reports containing HIV-indicators were extracted from DHIS2 for all care facilities in all counties in Kenya from 2011 to 2018. Data variables extracted included reporting rate, reporting timeliness, and HIV-indicator data elements per facility per year. 93,179 facility-records from 11,446 health facilities were extracted from year 2011 to 2018. *Van den Broeck *et al.*’s* framework, involving repeated cycles of a three-phase process (data screening, data diagnosis and data treatment), was employed semi-automatically within a generic five-step data-cleaning sequence, which was developed and applied in cleaning the extracted data. Various quality issues were identified, and Friedman analysis of variance conducted to examine differences in distribution of records with selected issues across eight years.

**Results:**

Facility-records with no data accounted for 50.23% and were removed. Of the remaining, 0.03% had over 100% in reporting rates. Of facility-records with reporting data, 0.66% and 0.46% were retained for voluntary medical male circumcision and blood safety programmatic area reports respectively, given that few facilities submitted data or offered these services. Distribution of facility-records with selected quality issues varied significantly by programmatic area (*p* < 0.001). The final clean dataset obtained was suitable to be used for subsequent secondary analyses.

**Conclusions:**

Comprehensive, systematic, and transparent reporting of cleaning-process is important for validity of the research studies as well as data utilization. The semi-automatic procedures used resulted in improved data quality for use in secondary analyses, which could not be secured by automated procedures solemnly.

## Background

Routine health information systems (RHIS) have been implemented in health facilities in many low-and middle-income countries (LMICs) for purposes such as facilitating data collection, management and utilization [1]. In order to ensure effectiveness of HIV programs, accurate, complete and timely monitoring and evaluation (M&E) data generated within these systems are paramount in decision-making such as resource allocation and advocacy [2]. Monitoring and Evaluation (M&E) plays a key role in planning of any national health program. De Lay et al. defined M&E as “acquiring, analyzing and making use of relevant, accurate, timely and affordable information from multiple sources for the purpose of program improvement [2].”

In order to provide strategic information needed for M&E activities in low- and middle-income countries (LMICs), reporting indicators have been highly advocated for use across many disease domains, with HIV indicators among the most common ones reported to national-level facilities in many countries [3–5]. As such, health facilities use pre-defined HIV-indicator forms to collect routine HIV-indicator data on various services provided within the facility, which are submitted to the national-level [6].

Over the years, national-level data aggregation systems, such as the District Health Information Software 2 (DHIS2) [7], have been widely adopted for use in collecting, aggregating and analyzing indicator data. DHIS2 has been implemented in over 40 LMICs with the health indicator data reported within the system used for national- and regional-level health-related decision-making, advocacy, and M&E [8]. Massive amounts of data have been collected within health information systems such as DHIS2 over the past several years, thus providing opportunities for secondary analyses [9]. However, these analyses can only be adequately conducted if the data extracted from systems such as DHIS2 are of high quality that is suitable for analyses [10].

Furthermore, data within health information systems such as DHIS2, are only as good as their quality, as this is salient for decision-making. As such, various approaches have been implemented within systems like DHIS2 to improve data quality. Some of these approaches include: (a) validation during data entry in order to ensure data are captured using the right formats and within pre-defined ranges and constraint; (b) user-defined validation rules; (c) automated outlier analysis functions such as standard deviation outlier analysis (identifies data values that are numerically extreme from the rest of the data), and minimum and maximum based outlier analysis (identifies data values outside the pre-set maximum and minimum values); and (d) automated calculations and reporting of data coverage and completeness [11]. WHO data quality tool has also been incorporated with DHIS2 to identify errors within the data in order to determine the next appropriate action [12]. Given that this tool is a relatively new addition to the DHIS2 applications, it is still being progressively improved and implemented in countries using DHIS2 [13].

Despite data quality approaches having been implemented within DHIS2, data quality issues remain a thorny problem, with some of the issues emanating from the facility level [14]. Real-life data like that found in DHIS2 are often “dirty” consisting of issues such as; incomplete, inconsistent, and duplicated data [15]. Failure to detect data quality issues and to clean these data can lead to inaccurate analyses outcomes [13]. Various studies have extracted data from DHIS2 for analyses [16–20]. Nonetheless, few studies attempt to explicitly disclose the data cleaning strategies used, resulting errors identified and the action taken [16–18]. In addition, some of these studies largely fail to exhaustively and systematically describe the steps used in data cleaning of the DHIS2 data before analyses are done [19, 20].

Ideally, data cleaning should be done systematically, and good data cleaning practice requires transparency and proper documentation of all procedures taken to clean the data [21, 22]. A closer and systematic look into data cleaning approaches, and a clear outlining of the distribution or characteristics of data quality issues encountered in DHIS2 could be instructive in informing approaches to further ensure higher quality data for analyses and decision-making. Further, employment of additional data cleaning steps will ensure that good quality data is available from the widely deployed DHIS2 system for use in accurate decision-making and knowledge generation.

In this study, data cleaning is approached as a process aimed at improving the quality of data for purposes of secondary analyses [21]. Data quality is a complex multidimensional concept. Wang and Strong categorized these dimensions as: intrinsic data quality, contextual data quality, representational and accessibility data quality [23]. Intrinsic data quality focuses on features that are inherent to data itself such as accuracy [23]. Contextual data quality focuses on features that are relevant in the context for the task for data use such as value-added, appropriate amount of data, and relevancy [23]. Representational and accessibility data quality highlights features that are salient within the role of the system such as interpretability, representational consistency, and accessibility [23]. Given that data quality can be subjective and dependent on context, various studies have specified context in relation to data quality [24–26]. Bolchini et al. specify context by tailoring data that are relevant for a given particular use case [27]. Bolchini et al. further posit that the process of separating noise (information not relevant to a specific task) to obtain only useful information, is not an easy task [27]. In this study, data cleaning is approached from a contextual standpoint, with the intention of retaining only relevant data for subsequent secondary analyses.

Therefore, the aim of this study is to report on the method and results of a systematic and replicable data cleaning approach employed on routine HIV-indicator data reports gathered within DHIS2 from 2011 to 2018 (8 year period), to be used for subsequent secondary analyses, using Kenya as a reference country case. This approach has specific applicability to the broadly implemented DHIS2 national reporting system. Our approach is guided by a conceptual data-cleaning framework, with a focus on uncovering data quality issues often missed by existing automated approaches. From our evaluation, we provide recommendations on extracting and cleaning data for analyses from DHIS2, which could be of benefit to M&E teams within Ministries of Health and by researchers to ensure high quality data for analyses and decision-making.

## Methods

### Data cleaning and data quality assessment approaches

Data cleaning is defined as “the process used to determine inaccurate, incomplete, or unreasonable data and then improving the quality through correction of detected errors and omissions” [28]. Data cleaning is essential to transform raw data into quality data for purposes such as analyses and data mining [29]. It is also an integral step in the knowledge discovery of data (KDD) process [30].

There exists various issues within the data, which necessitate cleaning in order to improve its quality [31–33]. An extensive body of work exists on how to clean data. Some of the approaches that can be employed include quantitative or qualitative methods. Quantitative approaches employ statistical methods, and are largely used to detect outliers [34–36]. On the other hand, qualitative techniques use patterns, constraints, and rules to detect errors [37]. These approaches can be applied within automated data cleaning tools such as ARKTOS, AJAX, FraQL, Potter’s Wheel and IntelliClean [33, 37, 38].

In addition, there are a number of frameworks used in assessment of data quality in health information systems, which can be utilized by countries with DHIS2. The Data Quality Review (DQR) tool developed in collaboration with WHO, Global Fund, Gavi and USAID/MEASURE Evaluation provides a standardized approach that aims at facilitating regular data quality checks [39]. Other tools for routine data quality assessments include the MEASURE Evaluation Routine Data Quality Assessment Tool (RDQA) [40] and WHO/IVB Immunization Data Quality Self-Assessment (DQS) [41].

Some of the data quality categories (intrinsic, contextual, representational and accessibility) [23], have been used in cleaning approaches as well as the data quality frameworks developed. A closer examination of the aforementioned approaches reveals focus on assessing intrinsic data quality aspects, which can be categorized further to syntactic quality (conformance to database rules) and semantic quality (correspondence or mapping to external phenomena) [42].

Moreover, while tools and approaches exist for data quality assessments as well as data cleaning, concerted efforts have been paced on assessment of health information system data quality [39, 40], as opposed to cleaning approaches for secondary analyses, which are largely dependent on the context for data use [24]. Wang and Strong posited the need for considering data quality with respect to context of the tasks, which can be a challenge as tasks and context vary by user needs [23]. Therefore, specifying the task and relevant features for the task, can be employed for contextual data quality [23, 43].

With this in mind and based on our knowledge, no standard consensus-based approach exists to ensure that replicable and rigorous data cleaning approaches and documentation are applied on extracted DHIS2 data to be used in secondary analyses. As such, ad hoc data cleaning approaches have been employed for the extracted data prior to analyses [16–18]. Moreover, whereas some studies provide brief documentation of data cleaning procedures used [19], others lack documentation, leaving the data cleaning approaches used undisclosed and behind-the-scenes [20]. Failure to disclose approaches used makes it difficult to replicate data cleaning procedures, and to ensure that all types of anomalies are systematically addressed prior to use of data for analysis and decision-making. Furthermore, the approach used in data extraction and cleaning affects the analysis results [21].

Oftentimes, specific approaches are applied based on the data set and the aims of the cleaning exercise [10, 44, 45]. Dziadkowiec et al. used Khan’s framework to clean data extracted from relational database of an Electronic Health Records (EHR) (10). In their approach, intrinsic data quality was in our view considered in data cleaning with focus on syntactic quality issues (such as conforming to integrity rules). Miao et al. proposed a data cleaning framework for activities that involve secondary analysis of an EHR [45], which in our view considered intrinsic data quality with focus on semantic quality (such as completeness and accuracy). Savik et al. approached data cleaning in our view from a contextual perspective, which entailed preparing the dataset that is appropriate for the intended analysis [44].

In this study, we approach data cleaning from a contextual perspective, whereby only data fit for subsequent analyses is retained. Based on our data set, our study’s data cleaning approach was informed by a conceptual data-cleaning framework proposed by Van den Broeck et al. [21]. Van den Broeck et al.’s framework was used because it provides a deliberate and systematic data cleaning guideline that is amenable to being tailored towards cleaning data extracted from DHIS2. This framework presents data cleaning as a three-phase process involving repeated cycles of data screening, data diagnosis, and data editing of suspected data abnormalities. The screening process involves identification of lacking or excess data, outliers and inconsistencies and strange patterns [21]. Diagnosis involves determination of errors or missing data and any true extremes and true normal [21]. Editing involves correction or deleting of any identified errors [21]. The various phases in Van den Broeck et al.’s framework have also been applied in various settings [46, 47]. Human-driven approaches complemented by automatic approaches were also used in the various data cleaning phases in thus study. Human-involvement in data cleaning has also been advocated in other studies [35].

### Study setting

This study was conducted in Kenya, a country in East Africa. Kenya adopted DHIS2 for use for its national reporting in 2011 [7]. The country has 47 administrative counties, and all the counties report a range of healthcare indicator data from care facilities and settings into the DHIS2 system. For the purposes of this study, we focused specifically on HIV-indicator data reported within Kenya’s DHIS2 system, given that these are the most comprehensively reported set of indicators into the system.

Kenya’s DHIS2 has enabled various quality mechanisms to deal with HIV data. Some of these include data validation rules, outlier analysis and minimum and maximum ranges, which have been implemented at the point of data entry. DHIS2 data quality tool is also an application that was included in DHIS2 to supplement the in-built data quality mechanisms [12]. Nonetheless it was not actively in use during our study period 2011–2018. The quality mechanisms as well as the DHIS2 quality tool consider intrinsic data quality aspects.

### Data cleaning process

Adapting the *Van den Broeck *et al.*’s* framework, a step-by-step approach was used during extraction and cleaning of the data from DHIS2. These steps are generic and can be replicated by others conducting robust data cleaning on DHIS2 for analyses. These steps are outlined below:

i **Step 1—**Outline the analyses or evaluation questions: Prior to applying the *Van den Broeck *et al.*’s* conceptual framework, it is important to identify the exact evaluations or analyses to be conducted, as this helps define the data cleaning exercise.
j **Step 2—**Description of data and study variables: This step is important for defining the needed data elements that will be used for the evaluation data set.
k **Step 3—**Create the data set: This step involves identifying the data needed and extracting data from relevant databases to generate the final data set. Oftentimes, development of this database might require combining data from different sources.
l **Step 4—**Apply the framework for data cleaning: During this step, the three data cleaning phases (screening, diagnosis, and treatment) in *Van den Broeck *et al.*’s* framework are applied on the data set created.
m **Step 5—**Analyze the data: This step provides a summary of the data quality issues discovered, the eliminated data after the treatment exercise, and the retained final data set on which analyses can then be done.

### Application of data cleaning process: Kenya HIV-indicator reporting case example

In this section, we present the application of the data cleaning sequence above using Kenya as case example. It is worth noting that in this study, the terms ‘programmatic area report’ and ‘report’ are used interchangeably as they contain the same meaning given that a report represents a programmatic area, and contains a number of indicators.

### Step 1: Outline the analyses or evaluation questions and goals

For this reference case, DHIS2 data had to undergo the data cleaning process prior to use of the data for an evaluation question on ‘Performance of health facilities at meeting the completeness and timeliness facility reporting requirements by the Kenyan Ministry of Health (MoH)’. The goal was to identify the best performing and poor performing health facilities at reporting within the country, based on completeness and timeliness in submitting their reports into DHIS2.

This study only attempts to clean the data for further subsequent analyses. Thus, the actual analyses and evaluation will be conducted using the final clean data in a separate study.

### Step 2: Description of data and study variables

HIV-indicator data in Kenya are reported into DHIS2 on a monthly basis by facilities offering HIV services using the MOH-mandated form called “*MOH 731- Comprehensive HIV/AIDS Facility Reporting Form*” (MOH731). As of 2011–2018, MOH 731 consisted of six programmatic areas representing six independent reports containing HIV-indicators to be reported [see Additional file 1]. The six reports and the number of indicators reported in each include: (1) HIV Counselling and Testing (HCT)—14 indicators; (2) Prevention of Mother-to-Child transmission (PMTCT)—40 indicators; (3) Care and Treatment (CrT)—65 indicators; (4) Voluntary Medical Male Circumcision (VMMC)—13 indicators; (5) Post-Exposure Prophylaxis (PEP)—14 indicators; and (6) Blood Safety (BS)—3 indicators.

Each facility offering HIV services is expected to submit reports with indicators every month based on the type(s) of services offered by that facility. Monthly due date for all reports are defined by the MoH, and the information on the expected number of reports per facility.

For our use case, we wanted to create a data set for secondary analyses, which was to determine performance of facilities at meeting the MoH reporting requirements (facility reporting completeness and timeliness of reporting). Hence, retain only facilities offering services for any of the six programmatic areas. Completeness in reporting by facilities within Kenya’s DHIS2 is measured as a continuous variable starting at 0% to 100% and identified within the system by a variable called ‘*Reporting Rate (RR)*’*.* The percentage RR is calculated automatically within DHIS2 as the actual number of reports submitted by each facility into DHIS2 divided by the expected number of reports from the facility multiplied by100 (Percentage RR = actual number of submitted reports/expected number of reports * 100). Given that MOH731 reports should be submitted by facilities on a monthly routine, the expected number of monthly reports per programmatic area per year is 12 (one report expected per month). It should be noted that this Reporting Rate calculation only looks at report submission and not the content within the reports. Given that facilities offering any of the HIV services are required to submit the full MOH731 form containing six programmatic area reports, zero (0) cases are reported for indicators where services are not provided, which appear as blank reports in DHIS2. As such, a report may be submitted as blank or have missing indicators but will be counted as complete (facility reporting completeness) simply because it was submitted. Timeliness is calculated based on whether the reports were submitted by the 15th day of the reporting month as set by the MoH. Timeliness is represented in DHIS2 as ‘*Reporting Rate on Time (RRT)*’ and is also calculated automatically. The percentage RRT for a facility is measured as a percentage of the actual number of reports submitted on time by the facility divided by the expected number of reports multiplied by 100 (Percentage RRT = actual number of reports submitted on time/expected number of reports * 100). Annual reports were therefore generated from DHIS2 consisting of percentage Reporting Rate and Reporting Rate on Time, which were extracted per facility, per year.

### Step 3: Create the data set

**Fig. 1:**
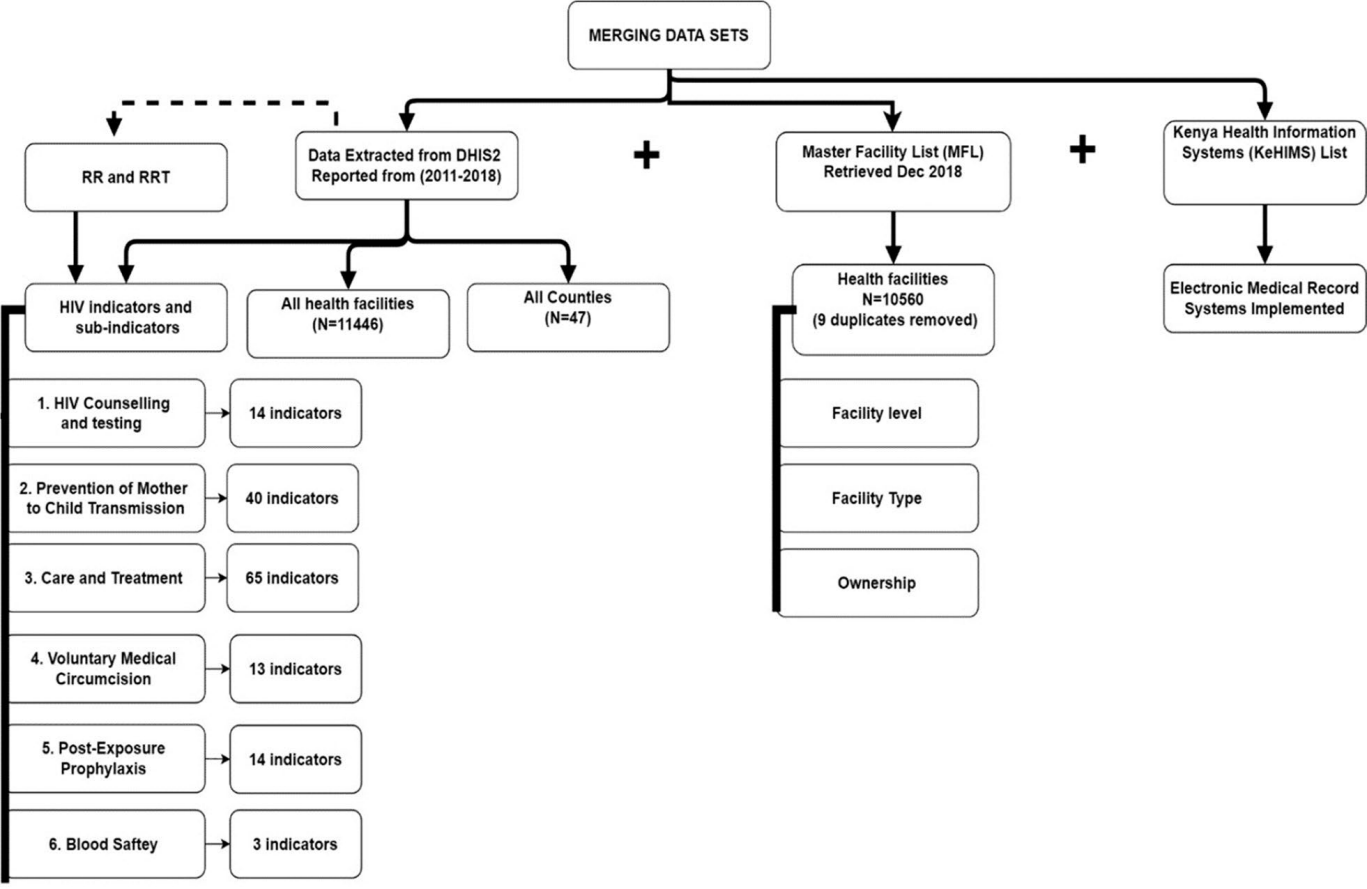
Creation of the evaluation data set

After obtaining Institutional Review and Ethics Committee (IREC) approval for this work, we set out to create our database from three data sources as outlined below:

1. *Data Extracted from DHIS2*: Two sets of data were extracted from DHIS2 to Microsoft Office Excel (version 2016). For the first data set, we extracted variables from DHIS2 for all HIV programmatic area reports submitted from all health facilities in all 47 counties in Kenya between the years 2011 and 2018, with variables grouped by year. Variables extracted from DHIS2 by year included: facility name, programmatic area report (e.g. Blood Safety), expected number of reports, actual number of submitted reports, actual number of reports submitted on time, cumulative Reporting Rate by year (calculated automatically in DHIS2) and cumulative Reporting Rate on Time by year (calculated automatically in DHIS2) [see Additional file 2]. The extracted data for Reporting Rate and Reporting Rate on Time constituted to the annual reports in the six programmatic areas for years 2011–2018, for the respective health facilities.
  For the second data set, we extracted the HIV-indicator data elements submitted within each annual programmatic area report by the health facilities for all the six programmatic areas for every year under evaluation [see Additional file 1].The annual report contained cumulative HIV-indicator data elements gathered in each programmatic area per facility, per year.
  In addition, extracting the aforementioned datasets from 2011 to 2018 resulted to repeated occurrence of the facility variable in the different years. For example, facilities registered in DHIS2 in 2011 will appear in subsequent years resulting to eight occurrences within the 8 years (2011–2018) per programmatic area report (e.g. Blood Safety). These resulted to a facility containing the following variables per row: facility name, year, percentage Reporting Rate, and percentage Reporting Rate on Time for the six programmatic area reports. In this study, the facility data per row was referred to as ‘facility record’.
2. *Facility Information*: We augmented the DHIS2 data with detailed facility information derived from Kenya Master Facility List (KMFL). This information included facility level (II–VI), facility type (such as dispensary, health center, medical clinic) and facility ownership (such as private practice, MoH-owned, owned by a non-governmental organization).
3. *Electronic Medical Record Status:* We used the Kenya Health Information Systems (KeHIMS) list, which contains electronic medical records (EMR) implemented in health facilities in Kenya, to incorporate information on whether the facility had an EMR or not. Information from these three sources were merged into a single data set as outlined in Fig. 1.

### Step 4: Application of the framework for data cleaning

**Fig. 2:**
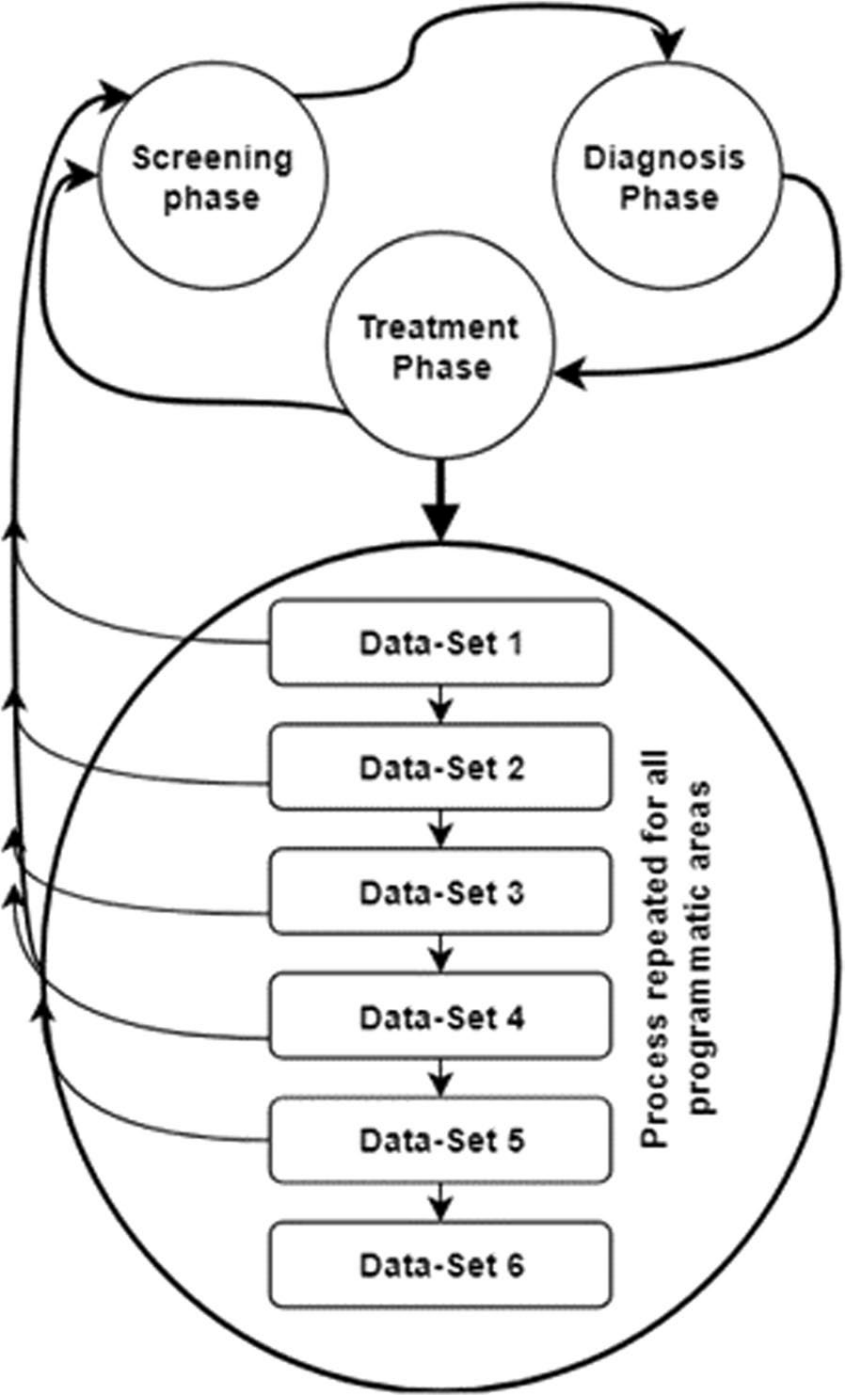
Repeated cycles of data cleaning

Figure 2 outlines the iterative cleaning process we applied adapting *Van den Broeck *et al.*’s* framework. Data cleaning involved repeated cycles of screening, diagnosis, and treatment of suspected data abnormalities, with each cycle resulting in a new data set. Details of the data cleaning process is outlined in Fig. 2.

- **Screening phase**

During the screening phase, five types of oddities need to be distinguished, namely: lack or excess of data; outlier (data falling outside the expected range); erroneous inliers; strange patterns in distributions and unexpected analysis results [21].

For determining errors, we used Reporting Rate and Reporting Rate on Time as key evaluation variables. Reporting Rate by itself only gives a sense of the proportion of expected reports submitted but does not evaluate whether exact HIV-indicator data elements are included within each report. To evaluate completion of HIV-indicator data elements within each of the programmatic area reports that were submitted, we created a new variable named ‘Cumulative Percent Completion (CPC)’. Using the annual report extracted for HIV-indicator data elements per facility, Cumulative Percent Completion was calculated by counting the number of non-blank values and dividing this by the total number of indicators for each programmatic area. As such, if a facility has reported on 10 out of 40 indicators in an annual report, it will have 25 percent on completeness. Therefore, Cumulative Percent Completion provides an aggregate annual summary of the proportion of expected indicator values that are completed within submitted reports. The results for Cumulative Percent Completion were then included as variables in the facility-records, described in step 3, section 1. This resulted to a facility-record containing the following variables per row: facility name, year, percentage Reporting Rate, percentage Reporting Rate on Time and Cumulative Percent Completion for the six programmatic areas.

b **Diagnostic phase**

The diagnostic phase enables clarification of the true nature of the worrisome data points, patterns, and statistics. Van den Broeck et al. posits possible diagnoses for each data point as: erroneous, true extreme, true normal or idiopathic (no diagnosis found, but data still suspected to having errors) [21]. We used a combination of Reporting Rate, Reporting Rate on Time and Cumulative Percent Completion to detect various types of situations (errors or no errors) for each facility per annual report (Table 1). Using the combination of Cumulative Percent Completion, Reporting Rate, and Reporting Rate on Time we were able to categorize the various types of situations to be used in diagnosis for every year a facility reported into DHIS2 (Table 1). In this table, “0*”* represents a situation where percentage is zero; “X” represents a situation where percentage is above zero; and “> 100%” represents a situation where percentage is more than 100. This data points were considered as erroneous records as the percentage reporting rate cannot go beyond 100 as this is not logically possible. Based on the values per each of the three variables, it was possible to diagnose the various issues within DHIS2 (Diagnosis Column).

For each programmatic area report (e.g. Blood Saftey) we categorized facilities by year and variables. All health facilities with an average Cumulative Percent Completion, Reporting Rate, and Reporting Rate on Time of zero (0) across all reports were identified as not having reported for the year and were henceforth excluded – as demonstrated by examples of Facility A and B in Table 2.

Beyond categorization of the various situations by report type, facility and year as defined above, errors related to duplicates were also identified using two scenarios. The first scenario of duplicates included a situation where health facilities had similar attributes such as year, name and county, with different data for Reporting Rate and Reporting Rate on Time. The second scenario of duplicates involves a situation where health facilities had similar attributes such as year, name and county, with similar data for Reporting Rate, and Reporting Rate on Time.

c **Treatment phase**

This is the final stage after screening and diagnosis, and entails deciding on the action point of the problematic records identified. Van den Broeck et al. limit the action points to correcting, deleting or leaving unchanged [21]. Based on the diagnosis illustrated in Table 1, facility-records in situation A-F were deleted hence excluded from the study. Duplicates identified in the scenarios mentioned were also excluded from the study. As such, for duplicates where health facilities had similar attributes such as year, name, and county, with different data for Reporting Rate, and Reporting Rate on Time, all entries were deleted. For duplicates where health facilities had similar attributes such as year, name, and county, with similar data for Reporting Rate, and Reporting Rate on Time, only one entry was deleted. Only reports in situation G and H were considered ideal for the final clean data set.

**Table 1:** Categorization of the various situations within DHIS2 and actions taken

Situation	CPC^a^	RR^b^	RRT^c^	Diagnosis	Action
A	0	0	0	Nothing was reported by facilities during this period, signifying that the facility does not report to DHIS2. This could be a true normal	Facility records excluded
B	0	X	X	Submitted reports might be on time, but are empty. Can result from programs wanting to have full MOH731 submission even though they do not offer services in all the 6 programmatic areas—hence submitting empty reports from non-required programmatic areas (Report is useless to decision-maker as it is empty)	Facility records excluded
C	0	X	0	Submitted reports are empty and not on time (Report is useless to decision-maker as it is empty and not on time)	Facility records excluded
D	X	0	0	No values present for RR and RRT. However, the reports are not empty	Facility records excluded
E	X	> 100%	X	Erroneous records as percentage RR cannot go beyond 100 as this is not logically possible	Facility records excluded
F	X	> 100%	> 100%	Erroneous records percentage RR and RRT cannot go beyond 100 as this is not logically possible	Facility records excluded
G	X	X	X	Reports submitted on time with relevant indicators included. Ideal situation	Facility records included
H	X	X	0	Submitted reports with data elements in them, but not submitted in a timely manner	Facility records included

^a^*CPC* cumulative percent completion, ^b^*RR* reporting rate, ^c^*RRT* reporting rate on time

**Table 2:** Example of sectional illustration of first data set containing facility records

Year	Organisation unit	CPC-HCT	RR-HCT	RRT-HCT	CPC-BS	RR-BS	RRT-BS	**	Avg-CPC	Avg-RR	Avg-RRT
2016	Facility A	0	0	0	0	0	0	0	0	0	0
2016	Facility B	0	0	0	0	0	0	0	0	0	0
2017	Facility C	10	90	80	100	90	80	0	50	60	50

*CPC* cumulative percentage completion, *RR-HCT* reporting rate HIV counselling and testing, *RRT* reporting rate on time, *BS* blood safety, *Avg* average, ** remaining four reports with the same variable sequence

### Step 5: Data analysis

The facility-records were then disaggregated to form six individual data sets representing each of the programmatic areas containing the following attributes: facility name, year, Cumulative Percent Completion, percentage Reporting Rate and percentage Reporting Rate on Time, as well as the augmented data on facility information and EMR status. The disaggregation was because facilities offer different services and do not necessarily report indicators for all the programmatic areas. SPSS was used to analyze the data using frequency distributions and cross tabulations in order to screen for duplication and outliers. Individual health facilities with frequencies of more than eight annual reports for a specific programmatic area were identified as duplicates. The basis for this is that the maximum annual reports per specific programmatic area for an individual health facility has to be eight, given that data was extracted within an eight-year period. From the cross tabulations, percentage Reporting Rate and percentage Reporting Rate on Time that were above 100% were identified as erroneous records.

After the multiple iterations of data cleaning as per Fig. 2, where erroneous data were removed by situation type (identified in Table 1), a final clean data set was available and brought forward to be used in a separate study for subsequent secondary analyses (which include answering the evaluation question in step 1). At the end of the data cleaning exercise, we determined the percentage distribution of the various situation types that resulted in the final data set. The percentages were calculated by dividing the number of facility-records in each situation type by the total facility-records in each programmatic area respectively, which was then multiplied by 100. As such, only data sets disaggregated into the six programmatic areas were included in the analysis. Using this analysis and descriptions from Table 1, we selected situation B, and situation D, in order to determine if there is a difference in distribution of facility records containing the selected situation types in the six programmatic areas across the 8 years (2011–2018).

This will enable comparing distribution of facility records by programmatic area categorized by situation B and situation D. The data contains related samples and is not normally distributed. Therefore, a Friedman analysis of variance (ANOVA) was conducted to examine if there is a difference in distribution of facility reports by programmatic area across all years N = 8 (2011–2018) for the selected situation types. As such, the variables analyzed include year, situation type, programmatic area, and unit of analysis include number of records in each situation type for a programmatic area. The distribution of facility-records was measured in all the six programmatic areas across the eight years and categorized by situation type. Wilcoxon Signed Rank Test were carried out as post hoc tests to compare significances in facility report distribution within the programmatic areas.

Below, we report on findings from the iterative data cleaning exercise and the resulting clean data set. The results further illustrate the value of the data cleaning exercise.

## Results

**Fig. 3:**
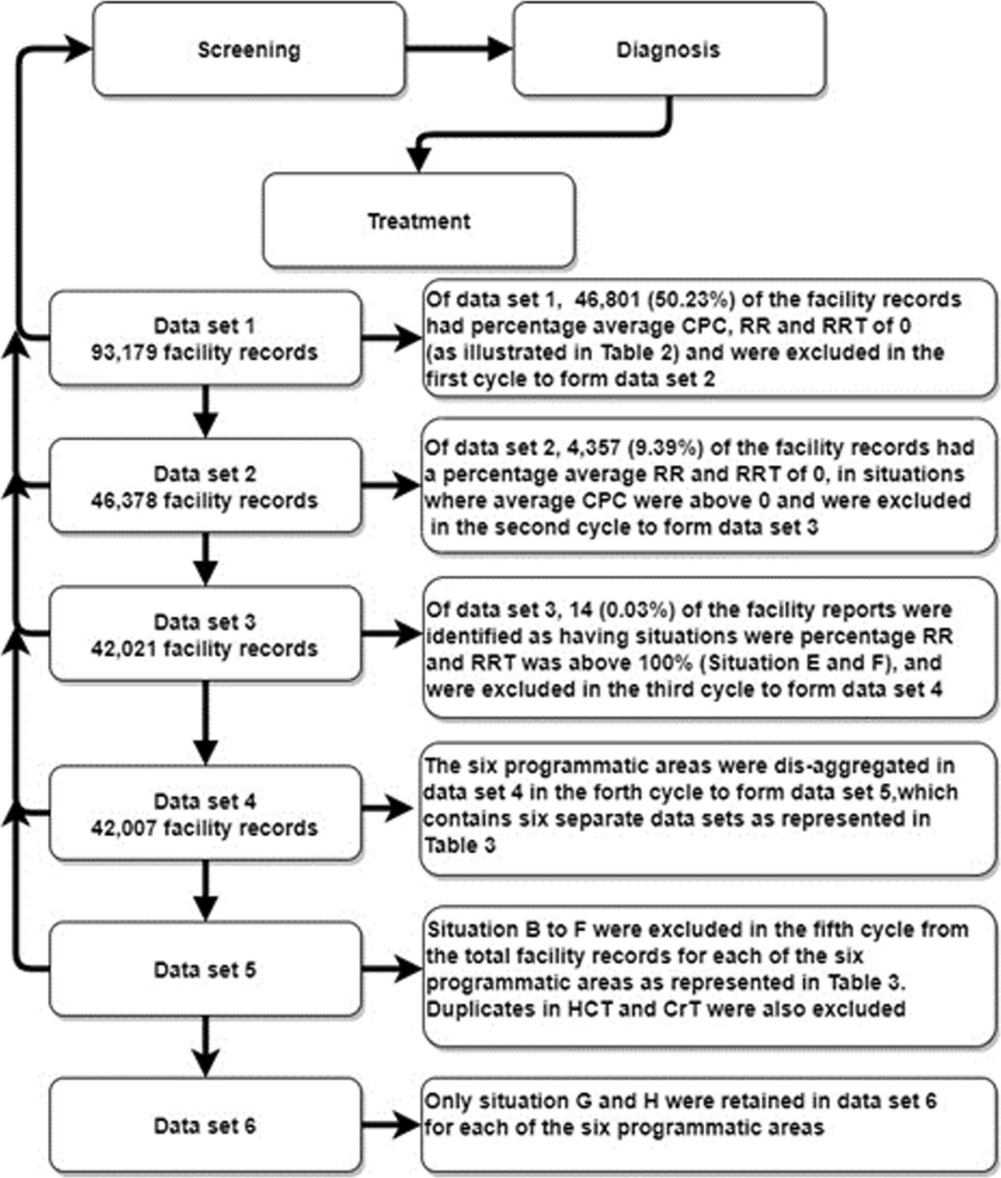
Data cleaning process

Figure 3 reports the various facility records at each cycle of the data cleaning process and the number (proportion) of excluded facility-records representing data with errors at each cycle.

The proportion of the resultant dataset after removal of the various types of errors from the facility records is represented in Table 3. A breakdown of reporting by facilities in descending order based on facility records retained after cleaning in dataset 4 is as follows; 93.98% were retained for HIV Counselling and Testing (HTC), 83.65% for Prevention of Mother to Child Transmission (PMTCT), 43.79% for Care and Treatment (CRT), 22.10% for Post Exposure Prophylaxis (PEP), 0.66% for Voluntary Medical Male Circumcision (VMMC), and 0.46% for Blood Safety (BS).

**Table 3:** Proportion of facility records (2011–2018) by programmatic area in the various situations based on facility records in dataset 4 (n = 42,007)

Situation	Facility records by programmatic area					
	HCT (%)	PMTC (%)	CrT (%)	VMMC (%)	PEP (%)	BS (%)
B(0XX)	2.68	6.15	1.32	2.81	18.04	1.70
C(0X0)	0.75	0.75	0.32	1.13	0.76	0.19
D(X00)	0.66	1.97	1.66	0.78	0.71	0.09
G(XXX)	92.44	81.52	42.60	0.63	21.82	0.45
H(XX0)	1.57	2.13	1.20	0.03	0.28	0.01
Duplicates	0.02	0.00	0.01	0.00	0.00	0.00
Total facility records (based on data set 4)	100.00	100.00	100.00	100.00	100.00	100.00
Total facility records removed	6.02	16.35	56.21	99.34	77.90	99.54
Total facility records retained	93.98	83.65	43.79	0.66	22.10	0.46

**Situation**-Detailed explanation of the various reporting situations within DHIS2 can be found in Table 1

**Fig. 4:**
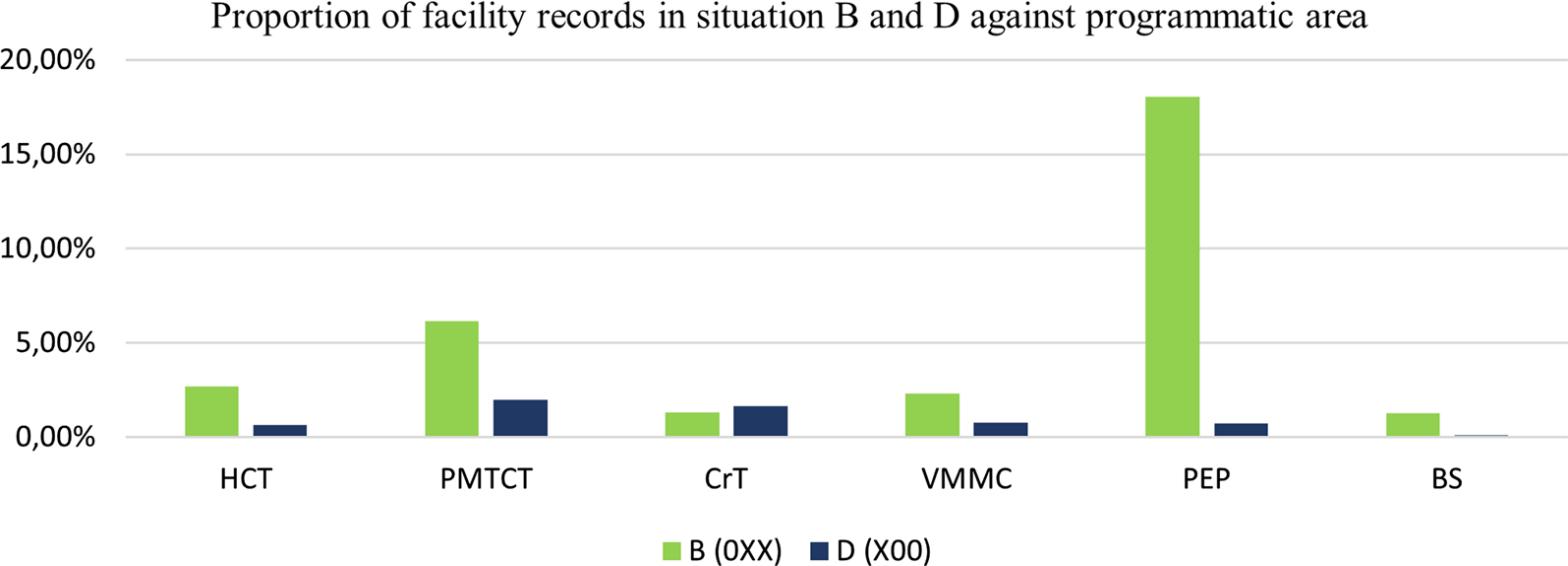
Distribution of facility records based on situation B (empty reports) and situation D against programmatic area

Situations where data was present in reports, but no values present for Reporting Rate and Reporting Rate on Time (Situation D); and scenarios with empty reports (Situation B) were analyzed (Fig. 4). This was in order to examine whether there are differences in distribution of facility records by programmatic area across the eight years, categorized by situation type. Most facilities submitted PEP empty reports (18.04%) based on data set 4 as shown in Fig. 4.

Overall Friedman Tests results for distribution of records with situation B and situation D in the various programmatic areas reveal statistically significant differences in facility record distribution (p = 0.001) across the eight years. Specific mean rank results categorized by error type are described in subsequent paragraphs.

Friedman Tests results for empty reports (Situation B) reveal that PEP had the highest mean rank of 6.00 compared to the other programmatic areas CT (3.50), PMTCT (4.88) CrT (2.00), VMMC (3.00), PEP and BS (1.63). Post hoc tests presented in Table 4 also reveal that PEP had higher distribution of facility records in situation B (0XX) in all the eight years.

**Table 4:** Results for Wilcoxon signed rank test for distribution of records in situation B

Situation B -Empty reports (0XX)			
Pairwise comparison by programmatic area	Wilcoxon signed ranks test (*P* value)	Wilcoxon signed ranks test (Z value)	Distribution of records in situation B based on pairwise comparison by programmatic area
PMTCT—HCT	0.012	− 2.521	Higher in PMTCT for 8 years
CrT—HCT	0.036	− 2.100	Lower in CrT for 6 years
PEP—HCT	0.012	− 2.521	Higher in PEP for 8 years
BS—HCT	0.012	− 2.524	Lower in BS for 8 years
CrT—PMTCT	0.017	− 2.521	Lower in CrT for 7 years
VMMC—PMTCT	0.012	− 2.521	Lower in VMMC for 8 years
PEP—PMTCT	0.012	− 2.521	Higher in PEP for 8 years
BS—PMTCT	0.012	− 2.524	Lower in BS for 8 years
VMMC—CrT	0.050	− 1.960	Higher in VMMC for 6 years
PEP—CrT	0.012	− 2.521	Higher in PEP for 8 years
PEP—VMMC	0.012	− 2.521	Higher in PEP for 8 years
BS—VMMC	0.012	− 2.524	Lower in BS for 8 years
BS—PEP	0.012	− 2.521	Lower in BS for 8 Years

*PMTCT* prevention of mother to child transmission, *HCT* HIV counselling and testing, *PEP* post-exposure prophylaxis, *BS* blood saftey, *CrT* care and treatment, *VMMC* voluntary medical male circumcision

Friedman Tests results for distribution of records with situation D (X00) reveal that PMTCT and CrT had the highest mean rank of 5.88 and 5.13 respectively compared to the other programmatic areas CT (3.00), VMMC (3.06), PEP (2.88) and BS (1.06). Post hoc tests presented in Table 5 reveal that PMTCT and CrT had higher distribution of facility records in situation D (X00) in all the 8 years.

**Table 5:** Results for Wilcoxon signed rank test for distribution of facility records in situation D (X00)

Situation D (X00)			
Pairwise comparison by programmatic area	Wilcoxon signed ranks test (*P* value)	Wilcoxon signed ranks test (Z value)	Distribution of records in situation D based on pairwise comparison by programmatic area
PMTCT—HCT	0.012	− 2.521	Higher in PMTCT for 8 years
CrT—HCT	0.012	− 2.521	Higher in CrT for 8 years
BS—HCT	0.012	− 2.524	Lower in BS for 8 years
VMMC—PMTCT	0.012	− 2.521	Lower in VMMC for 8 years
PEP—PMTCT	0.012	− 2.521	Lower in PEP for 8 years
BS—PMTCT	0.012	− 2.521	Lower in BS for 8 years
VMMC—CrT	0.012	− 2.524	Lower in VMMC for 8 years
PEP—CrT	0.012	− 2.527	Lower in PEP for 8 years
BS—CrT	0.012	− 2.524	Lower in BS for 8 years
BS—VMMC	0.018	− 2.375	Lower in BS for 8 years
BS—PEP	0.012	− 2.524	Lower in BS for 8 years

*PMTCT* prevention of mother to child transmission, *HCT* HIV counselling and testing, *CrT* care and treatment, *PEP* post-exposure prophylaxis, *BS* blood safety, *VMMC* Voluntary Medical Male Circumcision

## Discussion

Systematic data cleaning approaches are salient in identifying and sorting issues within the data resulting to a clean data set that can be used for analyses and decision-making [21]. This study presents the methods and results of systematic and replicable data cleaning approach employed on routine HIV-indicator data reports in preparation for secondary analyses.

For data stored in DHIS2, this study assumed that the inbuilt data quality mechanisms dealt with the pre-defined syntactical data quality aspects such as validation rules. As such, the contextual approach to data cleaning was employed on extracted data from DHIS2 with the aim of distinguishing noise (data that are not relevant for intended use or of poor quality), from relevant data as presented by the various situations in Table 1. As demonstrated in this study, identifying various issues within the data may require a human-driven approach as inbuilt data quality checking mechanisms within systems may not have the benefit of a particular knowledge. Furthermore, these human augmented processes also facilitated diagnosis of the different issues, which would have gone unidentified. For instance, our domain knowledge about health facility HIV reporting enabled us to identify the various situations described in Table 1. This entailed examining more than one column at a time of manually integrated databases and using the domain knowledge in making decisions on actions to take on the data set (treatment phase). Similarly, Maina et al. also used domain knowledge on maternal and child bearing programmes in adjusting for incomplete reporting [48].In addition, descriptive statistics such as use of cross tabulations and frequency counts complemented the human-driven processes, in order to identify issue within the data such as erroneous records (screening phase).

The use of Cumulative Percent Completeness (CPC) in this study facilitated screening and diagnosis of problematic issues highlighted in similar studies that are consistent with our findings. These include identifying and dealing with non-reporting facilities (situation A), and non-service providing facilities (situation B and C) in a data set [19, 48]. This comes about as some of the reports extracted contain blanks, as DHIS2 is unable to record zeros as identified in other studies [16–19, 49]. As such, DHIS2 is unable to distinguish between missing values and true zero values. Therefore, facilities containing such records either are assumed to not be providing the particular service in question or are non-reporting facilities (providing services but not reporting or not expected to provide reports).

In most cases, such records are often excluded from the analyses [19, 48], as was the approach applied in this study. Furthermore, non-service providing facilities were excluded on the basis that they may provide inaccurate analyses for the evaluation question described in step1. This is on the basis that analyses may portray facilities as having good performance in facility reporting completeness and timeliness; hence give a wrong impression as no services were provided in a particular programmatic area (situation B and C). As such, even though a report was submitted on time by a facility, it will not be of benefit to a decision-maker as the report has no indicators (is empty). Nonetheless, it is worth noting that reporting facilities considered to be providing HIV services but had zero percent in timeliness were retained as these records were necessary for the subsequent analyses.

Maiga et al. posit that non-reporting facilities are often assumed not to be providing any services given that reporting rates are often ignored in analyses [13]. With this in mind, this study considered various factors prior to exclusion of non-reporting facility records. This include identifying whether there were any successful report submissions in the entire year, and whether the submitted reports contained any data in the entire year. Therefore, facilities with records that did not meet this criteria (situation A, B, and C) were considered as non-service providing in the respective programmatic areas.

Further still, another finding consistent with similar studies is that of identifying and dealing with incomplete reporting, which can be viewed from various perspectives. This can include a situation where a report for a service provided has been successfully submitted but is incomplete [17, 19, 48]; or missing reports (expected reports have not been submitted consistently for all 12 months), hence making it difficult to identify whether services were provided or not, in months were reports were missing [48]. Whereas some studies retain these facility records, others opt to make adjustments for incomplete reporting. Maiga et al. posit that these adjustments need to be made in a transparent manner when creating the new data set with no modifications made on the underlying reported data [13].

In this study, all facility records were included (situation G and H) irrespective of incomplete reporting, which was similar to the approach taken by Thawer et al. [19]. On the other hand, Maina et al. opted to adjust for incomplete reporting, apart from where missing reports were considered an indication that no services were provided [48]. Furthermore, a number of studies in DHIS2 have identified duplicate records [16, 18, 19], with removal or exclusion as the common action undertaken to prepare the data set for analyses. These findings thus demonstrate duplication as a prevalent issue within DHIS2 [16, 18, 19, 49].

Whereas studies using DHIS2 data have found it necessary to clean the extracted data prior to analyses [16, 18, 19], transparent and systematic approaches are still lacking in literature [20]. Given that contexts were data is being used vary, there is no one-size fits all solution to data cleaning, considering the many existing approaches as well as the subjective component of data quality [25, 26]. As such, transparent and systematic documentation of procedures is valuable as it also increases the validity in research [21]. Moreover, existing literature advocates the need for clear and transparent description of data set creation and data cleaning methods [9, 21, 22]. Therefore, the generic five-step approach developed in this study is a step toward the right direction as it provides a systematic sequence that can be adopted for cleaning data extracted from DHIS2.

In addition, the statistical analysis employed such as non-parametric tests provide an overview of distribution of facility records containing quality issues within the various programmatic areas, hence necessitating need for further investigations where necessary. These statistics also provided a picture of the most reported programmatic areas, which contain data within their reports.

Moreover, as revealed in the screening, diagnosis and treatment phases presented in this paper, data cleaning process can be time consuming. Real-world data such as the DHIS2 data and merging of real-world data sets as shown in this paper may be noisy, inconsistent and incomplete. In the treatment stage, we present the actions taken to ensure that only meaningful data is included for subsequent analysis. Data cleaning also resulted to a smaller data set than the original as demonstrated in the results [29]. As such, the final clean data set obtained in this study is more suitable for its intended use than in its original form.

A limitation in this study was inability to determine the causality of some of the issues encountered. Whereas quality issues are in part attributed to insufficient skills or data entry errors committed at the facility level [14], some of the issues encountered from our findings (such as duplication, situation E and F) are assumed to be stemming from within the system. Nonetheless, there is need for further investigation on causality. In addition, given that situation D was identified as a result of merging two data sets extracted from DHIS2, it was expected that if reports contain indicator data, then their respective Reporting Rate and Reporting Rate on Time should be recorded. Nonetheless, it was also not possible within the confines of this study to identify the causality for situation D. As such, further investigations are also required.

In addition, there are also limitations with human augmented procedures as human is to error especially when dealing with extremely large data sets as posited by other studies [24]. Moreover, data cleaning for large data sets can also be time consuming. Nonetheless, identifying and understanding issues within the data using a human-driven approach provides better perspective prior to developing automatic procedures, which can then detect the identified issues. Therefore, there is need for developing automated procedures or tools for purposes of detecting and handling the different situation types in Table 1.

DHIS2 incorporated a quality tool, which used a similar concept as that used in calculating Cumulative Percent Completion in this study, to flag facilities with more than 10 percent zero or missing values in the annual report [12]. Based on this, we recommend that facilities with 100 percent zero or missing values also be flagged in the annual report in order to identify empty reports, as well situation where Reporting Rate on Time is zero in the annual report. Further still automated statistical procedures can be developed within the system to perform various analyses such as calculating the number of empty reports submitted by a facility for a sought period of time, per programmatic area. This could provide beneficial practical implications such as enabling decision-makers to understand the frequency of provision of certain services among the six programmatic areas within a particular period among health facilities. We also recommend for measures to be established within DHIS2 implementations to ensure that cases reported as zero appear in DHIS2.

Such findings could be used to improve the quality of reporting. Automatic procedures should also be accompanied by data visualizations, and analyses, integrated within the iterative process in order to provide insights [35]. In addition, user engagement in development of automatic procedures and actively training users in identifying and discovering various issues within the data may contribute to better quality of data [35, 37].

## Conclusion

Comprehensive, transparent and systematic reporting of cleaning process is important for validity of the research studies [21]. The data cleaning included in this article was semi-automatic. It complemented the automatic procedures and resulted in improved data quality for data use in secondary analyses, which could not be secured by the automated procedures solemnly. In addition, based on our knowledge, this was the first systematic attempt to transparently report on the developed and applied data cleaning procedures for HIV-indicator data reporting in DHIS2 in Kenya. Furthermore, more robust and systematic data cleaning processes should be integrated to current inbuilt DHIS2 data quality mechanisms to ensure highest quality data.

## Supplementary information

**Additional file 1**. Programmatic areas (reports) with respective indicators as per MOH 731- Comprehensive HIV/AIDS Facility Reporting Form extracted from DHIS2.

**Additional file 2**. Facility report submission data extracted from DHIS2.

## Data Availability

The data sets generated during the current study are available in the national District Health Information Software 2 online database, https://hiskenya.org/.

## Abbreviations

BS: Blood safety
CPC: Cumulative percent completion
CrT: Care and treatment
DHIS2: District Health Information System Version 2
EMR: Electronic medical record
HIV: Human immunodeficiency virus
HCT: HIV counselling and testing
KeHMS: Kenya Health Management System
KMFL: Kenya Master Facility List
LMICs: Low-and middle-income countries
MOH: Ministry of Health
NGO: Non-Governmental Organization
PEP: Post-exposure prophylaxis
PMTCT: Prevention of mother to child transmission
RHIS: Routine health information systems
RR: Reporting rate
RRT: Reporting rate on time
VMMC: Voluntary Medical Male Circumcision
