# Supplementary material for: Data cleaning process for HIV-indicator data extracted from DHIS2 national reporting system: a case study of Kenya
Source: BMC Med Inform Decis Mak. 2020 Nov 13;20:293. doi: 10.1186/s12911-020-01315-7 (PMC7664027; doi:10.1186/s12911-020-01315-7)
Supplement: Supplementary file 1 — Additional file 1. Programmatic areas (reports) with respective indicators as per MOH 731- Comprehensive HIV/AIDS Facility Reporting Form extracted from DHIS2. [file 12911_2020_1315_MOESM1_ESM.docx]

**Additional file 1: Programmatic areas (reports) with respective indicators as per MOH 731- Comprehensive HIV/AIDS Facility Reporting Form extracted from DHIS2**

Indicators in each of the programmatic areas (reports) were extracted per facility per year.

|  | **MOH 731-1 HIV Counselling And Testing** |  | **MOH 731-3 Care and Treatment** |  | **MOH 731-4 Voluntary Male Circumcision** |
| --- | --- | --- | --- | --- | --- |
| 1 | Concordant Couples Receiving Results (Couples Only) | 1 | ART Net Cohort at 12 months Survival and Retention on ART | 1 | Circumcised 0-14 yrs |
| 2 | Couples Testing | 2 | Currently on ART - Female Below 15 years | 2 | Circumcised 15-24 yrs |
| 3 | Discordant Couples Receiving Results (Couples Only) | 3 | Currently on ART - Female above 15 years | 3 | Circumcised 25 yrs and Above |
| 4 | Female 15-24yrs Receiving HIV + Results | 4 | Currently on ART - Male above 15 years | 4 | During - AE(s) Moderate Adverse Events (Circumcision) |
| 5 | Female above 25yrs Receiving HIV + Results | 5 | Currently on ART - Male below 15 years | 5 | During - AE(s) Severe Adverse Events (Circumcision) |
| 6 | Female under 15yrs Receiving HIV + Results | 6 | Currently on ART - below 1 year | 6 | Negative -HIV Status (at circumcision) |
| 7 | First Testing HIV | 7 | Female above 15yrs Starting on ART | 7 | Positive -HIV Status (at circumcision) |
| 8 | Male 15-24yrs Receiving HIV + Results | 8 | Female 15 years and older screened for TB | 8 | Post - AE(s) Moderate Adverse Events (Circumcision) |
| 9 | Male above 25yrs Receiving HIV + Results | 9 | Female above 15yrs Revisit on ART | 9 | Post - AE(s) Severe Adverse Events (Circumcision) |
| 10 | Male under 15yrs Receiving HIV + Results | 10 | Female above 15yrs Enrolled in Care | 10 | Total AE During |
| 11 | Outreach Testing HIV | 11 | Female above 15yrs Ever on ART | 11 | Total AE Post |
| 12 | Repeat Testing HIV | 12 | Female under 15 years screened for TB | 12 | Total Circumcised |
| 13 | Static Testing HIV (Health Facility) | 13 | Female under 15yrs Revisit on ART | 13 | Unknown -HIV Status (at circumcision) |
| 14 | Total Tested HIV | 14 | Female under 15yrs Currently in Care |  | **MOH 731-5 Post-Exposure Prophylaxis** |
|  | **MOH 731-2 Prevention of Mother To Child Transmission** | 15 | Female under 15yrs Enrolled in Care | 1 | Occupational Female Provided with Prophylaxis |
| 1 | Antenatal Positive to HIV Test | 16 | Female under 15yrs Ever on ART | 2 | Occupational Female Type of Exposure |
| 2 | Antenatal Testing for HIV | 17 | Female under 15yrs Starting on ART | 3 | Occupational Male Provided with Prophylaxis |
| 3 | Assessed Eligibility in ANC | 18 | HIV Care visit Scheduled | 4 | Occupational Male Type of Exposure |
| 4 | Assessed for eligibility in 1st ANC - CD4 | 19 | HIV Care visit- unscheduled | 5 | Other reasons Female Type of Exposure |
| 5 | Assessed for eligibility in 1st ANC - WHO Staging done | 20 | HIV Care visits Females (18 years and older) | 6 | Other reasons Male Type of Exposure |
| 6 | BF (at 12 months) Infant Feeding | 21 | HIV Currently in Care - Total | 7 | Other reasons – female -Provided with Prophylaxis |
| 7 | Discordant Couples Partner Involvement | 22 | HIV Currently in Care - above 15yrs Female | 8 | Other reasons – male - Provided with Prophylaxis |
| 8 | EBF (6 months) Infant Feeding | 23 | HIV Exposed Infant (Eligible for CTX 2 months) | 9 | Sexual assault Female Provided with Prophylaxis |
| 9 | ERF (6 months) Infant Feeding | 24 | HIV Exposed Infant (within 2 months) on Cotrimoxazole Prophylaxis | 10 | Sexual assault Female Type of Exposure |
| 10 | Issued in ANC (Infant ARV prophylaxis) | 25 | Male above 15yrs & Older Enrolled in Care | 11 | Sexual assault Male Provided with Prophylaxis |
| 11 | Known positive status (at entry into ANC) | 26 | Male above 15yrs Ever on ART | 12 | Sexual assault Male Type of Exposure |
| 12 | Labour and Delivery (Infant ARV prophylaxis) | 27 | Male 15 years and older screened for TB | 13 | Total PEP |
| 13 | Labour and Delivery Postive to HIV Test | 28 | Male Below 15 years screened for TB | 14 | Total type of Exposure |
| 14 | Labour and Delivery Testing for HIV | 29 | Male above 15yrs Revisit on ART |  | **MOH 731-6 Blood Safety** |
| 15 | MF (6 months) Infant Feeding | 30 | Male above 15yrs Currently in Care | 1 | Blood units reactive to HIV |
| 16 | Male partners tested -( ANC/L&D) | 31 | Male above 15yrs Starting on ART | 2 | Blood units screened for TTIs |
| 17 | Not BF (12 months) Infant Feeding | 32 | Male under 15yrs Ever on ART | 3 | Donated blood units |
| 18 | Not Known Infant Feeding (12 months) | 33 | Male under 15yrs Revisit on ART |  |  |
| 19 | PCR (3 to 8 months) Confirmed Infant Test Results Positive | 34 | Male under 15yrs Currently in Care |  |  |
| 20 | PCR (9 to 12 months) Confirmed Infant Test Results Positive | 35 | Male under 15yrs Enrolled in Care |  |  |
| 21 | PCR (by 2 months) Confirmed Infant Test Results Positive | 36 | Male under 15yrs Starting on ART |  |  |
| 22 | PCR (from 9 to 12 months) Infant Testing (Initial test only) | 37 | Modern contraceptive methods |  |  |
| 23 | PCR (from3 to 8 months) Infant Testing (Initial test only) | 38 | Number started on Isoniazid Preventive Therapy Male < 15 yrs |  |  |
| 24 | PCR (within 2 months) Infant Testing (Initial test only) | 39 | Number started on Isoniazid Preventive Therapy Male > 15 yrs |  |  |
| 25 | PNC (<72hrs) (Infant ARV prophylaxis) | 40 | Number started on Isoniazid Preventive Therapy female < 15 yrs |  |  |
| 26 | Postnatal (within 72hrs) Postive to HIV Test | 41 | Number started on Isoniazid Preventive Therapy female > 15 yrs |  |  |
| 27 | Postnatal (within 72hrs) Testing for HIV | 42 | On 2nd Line (or higher) at 12 months Survival and Retention on ART |  |  |
| 28 | Prophylaxis - (AZT+SdNVP) | 43 | On CTX 15 y and Older Male |  |  |
| 29 | Prophylaxis - interrupted HAART | 44 | On CTX 15 yrs and Older Female |  |  |
| 30 | Prophylaxis – HAART | 45 | On CTX Below 15 yrs Female |  |  |
| 31 | Prophylaxis-NVP Only | 46 | On CTX Below 15 yrs Male |  |  |
| 32 | Serology (from 9 to 12 months) Infant Testing (Initial test only) | 47 | On Original 1st Line at 12 months Survival and Retention on ART |  |  |
| 33 | Total Confirmed Positive Infant test result by PCR | 48 | On alternative 1st Line at 12 months Survival and Retention on ART |  |  |
| 34 | Total Exposed 12 months | 49 | Pregnant women Starting on ART |  |  |
| 35 | Total Exposed aged 6 months | 50 | Provided with condoms |  |  |
| 36 | Total HEI tested by 12 months | 51 | Screened for cervical cancer (females 18 years and older) |  |  |
| 37 | Total Infants Issued Prophylaxis | 52 | TB Patient Starting on ART |  |  |
| 38 | Total PMTCT prophylaxis | 53 | Total Enrolled in Care |  |  |
| 39 | Total Positive (PMTCT) | 54 | Total Ever on ART |  |  |
| 40 | Total Tested (PMTCT) | 55 | Total HIV Care visit |  |  |
|  |  | 56 | Total Revisit on ART |  |  |
|  |  | 57 | Total Screened for TB |  |  |
|  |  | 58 | Total Starting on ART |  |  |
|  |  | 59 | Total currently on ART |  |  |
|  |  | 60 | Total on CTX |  |  |
|  |  | 61 | Total on therapy at 12 months |  |  |
|  |  | 62 | Under 1yr Revisit on ART |  |  |
|  |  | 63 | Under 1yr Currently in Care |  |  |
|  |  | 64 | Under 1yr Enrolled in Care |  |  |
|  |  | 65 | Under 1yr Starting on ART |  |  |
|  |  |  |  |  |  |
