# Supplementary material for: Data cleaning process for HIV-indicator data extracted from DHIS2 national reporting system: a case study of Kenya
Source: BMC Med Inform Decis Mak. 2020 Nov 13;20:293. doi: 10.1186/s12911-020-01315-7 (PMC7664027; doi:10.1186/s12911-020-01315-7)
Supplement: Supplementary file 2 — Additional file 2. Facility report submission data extracted from DHIS2. [file 12911_2020_1315_MOESM2_ESM.docx]

| Period (year) |
| --- |
| Organisation unit (health facility) |
| MOH 731-1 HIV Counselling And Testing Actual reports on time |
| MOH 731-1 HIV Counselling And Testing Actual reports |
| MOH 731-1 HIV Counselling And Testing Expected reports |
| **MOH 731-1 HIV Counselling And Testing Reporting rate** |
| **MOH 731-1 HIV Counselling And Testing Reporting rate on time** |
| MOH 731-2 PMTCT Actual reports on time |
| MOH 731-2 PMTCT Actual reports |
| MOH 731-2 PMTCT Expected reports |
| **MOH 731-2 PMTCT Reporting rate** |
| **MOH 731-2 PMTCT Reporting rate on time** |
| MOH 731-3 Care and Treatment Actual reports on time |
| MOH 731-3 Care and Treatment Actual reports |
| MOH 731-3 Care and Treatment Expected reports |
| **MOH 731-3 Care and Treatment Reporting rate** |
| **MOH 731-3 Care and Treatment Reporting rate on time** |
| MOH 731-4 Voluntary Male Circumcision Actual reports on time |
| MOH 731-4 Voluntary Male Circumcision Actual reports |
| MOH 731-4 Voluntary Male Circumcision Expected reports |
| **MOH 731-4 Voluntary Male Circumcision Reporting rate** |
| **MOH 731-4 Voluntary Male Circumcision Reporting rate on time** |
| MOH 731-5 Post-Exposure Prophylaxis Actual reports on time |
| MOH 731-5 Post-Exposure Prophylaxis Actual reports |
| MOH 731-5 Post-Exposure Prophylaxis Expected reports |
| **MOH 731-5 Post-Exposure Prophylaxis Reporting rate** |
| **MOH 731-5 Post-Exposure Prophylaxis Reporting rate on time** |
| MOH 731-6 Blood Safety Actual reports on time |
| MOH 731-6 Blood Safety Actual reports |
| MOH 731-6 Blood Safety Expected reports |
| **MOH 731-6 Blood Safety Reporting rate** |
| **MOH 731-6 Blood Safety Reporting rate on time** |

**Additional file 2: Facility report submission data extracted from DHIS2.**

The following variables were extracted. Only the highlighted variables were included in the dataset as these consisted of the automatically calculated percentage Reporting Rate (RR) and Reporting Rate on time (RRT).

These variables were extracted per facility per year (2011 to 2018).
